# Neurobiological underpinnings of cyberbullying: A pilot functional magnetic resonance imaging study

**DOI:** 10.1002/hbm.24890

**Published:** 2019-12-04

**Authors:** Larisa T. McLoughlin, Zack Shan, Kathryn M. Broadhouse, Natalie Winks, Gabrielle Simcock, Jim Lagopoulos, Daniel F. Hermens

**Affiliations:** ^1^ Sunshine Coast Mind and Neuroscience Thompson Institute (SCMNTI) University of the Sunshine Coast, 12 Innovation Parkway, Birtinya 4575 Queensland

**Keywords:** brain, cyberbullying, Cyberbullying Picture Series, cyberbystander, function magnetic resonance imaging, neurobiology, protocol, scenarios

## Abstract

There is a dearth of research that has investigated the neural correlates of cyberbullying, using task‐based functional magnetic resonance imaging (fMRI) and, specifically, in a real‐time context such as observing cyberbullying scenarios. This article presents pilot data from a novel protocol designed to undertake such research with the overall aim being to elucidate the neurobiological underpinnings of cyberbullying via task‐based fMRI (tb‐fMRI)) in passive cyberbystanders. Young adults (*N* = 32, 18 to 25 years old) viewed six negative (cyberbullying) and six neutral stimuli from the Cyberbullying Picture Series (CyPicS) while undergoing tb‐fMRI. Our results revealed 12 clusters of significantly greater blood‐oxygenation‐level‐dependent (BOLD) responses (family wise error corrected *p*_*FWE*_ < .05) in participants when viewing cyberbullying stimuli compared to neutral stimuli, across a distributed network of regions including left and right middle temporal gyrus, default mode network hubs, left and right posterior cerebellum/vermis, and putamen. Further analysis also revealed greater BOLD response in females compared to males, as well as in those with no prior experience of cyberbullying compared to those with prior experience (despite gender), when viewing the cyberbullying stimuli compared to the neutral stimuli. These results bring us closer to understanding the neurobiological underpinnings that may be associated with cybervictim/bully status and outcomes.

## 1 INTRODUCTION

Cyberbullying is defined as an aggressive, repeated, intentional act carried out on an individual using electronic forms (Smith et al., 2008) and can have serious impacts on mental health (Fahy et al., 2016; Le et al., 2017; McLoughlin, Spears, & Taddeo, 2018; McLoughlin, Spears, Taddeo, & Hermens, 2019). Victims of cyberbullying report significantly more social difficulties, higher levels of anxiety and depression, and are more likely to suffer suicidal ideation (Kowalski, Giumetti, Schroeder, & Lattanner, 2014; van Geel, Vedder, & Tanilon, 2014) than victims of traditional bullying. Given the recognized links between cyberbullying and negative mental health outcomes, it is imperative that research regarding cyberbullying and the brain be conducted.

Limited research has specifically examined the links between cyberbullying and brain development in young people (i.e., adolescents and young adults), particularly regarding the use of neuroimaging. Despite this, emerging functional magnetic resonance imaging (fMRI) research indicates that online social interactions are associated with similar structural correlates and patterns of brain activity to those observed in the context of real‐world relationships (Lamblin, Murawski, Whittle, & Fornito, 2017). In this regard, young people respond in a similar manner to positive feedback online (such as “likes” on their photos or updates) as they would in a face‐to‐face conversation (Sherman, Payton, Hernandez, Greenfield, & Dapretto, 2016). Notably, a review on traditional bullying in young people found that the brain experiences peer victimization in a similar way to physical pain and that these experiences can become biologically embedded in the physiology of the developing person, thereby increasing their risk of developing mental health problems (Vaillancourt, Hymel, & McDougall, 2013).

Other researchers have reported neural mechanisms associated with conduct disorders, antisocial behavior, and empathy in children and indicate that serotonergic and stress‐regulating mechanisms may explain individual differences in antisocial behavior (Blair, 2013; van Goozen, Fairchild, Snoek, & Harold, 2007; Viding, McCrory, Blakemore, & Frederickson, 2011). Similarly, impairment in the neural circuits involved in emotion processing has been linked to a propensity towards aggressive behavior, and such behavior is associated with abnormalities in neural processes that promote both the inhibitory control of and the flexible adaptation of behavior (Sterzer & Stadler, 2009). As such, research on neurobiological factors associated with conduct disorders and aggression have a key role to play in informing how schools manage traditional bullying, regarding which students are most at risk of bullying behaviors and which are more likely to benefit from interventions. However, research regarding cyberbullying and neurobiology is limited.

One study which specifically examined cyberbullying in young people aged 11 to 18 years, found that patterns of cortisol release (a hormone associated with stress) and perceived stress were related to roles in cyberbullying, with cybervictims (those who have only ever been a victim of cyberbullying) and cyberbully‐victims (those who have been both a bully and a victim of cyberbullying) exhibiting higher cortisol secretion levels and greater perceived stress compared to cyberbullies and cyberbystanders (González‐Cabrera, Calvete, León‐Mejía, Pérez‐Sancho, & Peinado, 2017). More specifically, the lowest cortisol secretion was observed in serious cyberbullies and cyberbullying victimization was significantly related to an elevated profile of cortisol secretion (González‐Cabrera et al., 2017). These findings suggest that there is a biological basis, possibly related to physiological stress, that may distinguish different roles or “types” in cyberbullying.

### 1.1 Cyberbystanders

While there has been substantial research on the role (i.e., behaviors) of cyberbystanders, very limited research has employed real‐time scenarios to measure young people's responses and neurobiological reactions to an incident, with even less research examining how the brain responds to witnessing cyberbullying. Online bystanders are much more complicated than bystanders “offline,” as cyberbystanders not only have the power to contribute to bullying others by forwarding cyberbullying posts to their friends or others (an infinite number of times) but they could be with the cyberbully when the post is made, with the cybervictim when it is received, or witness the sharing and forwarding of bullying posts (Kowalski, Limber, Limber, & Agatston, 2012; Menesini & Nocentini, 2009; Smith, 2014).

Research into cyberbystanders investigating 1,412 adolescents aged 10 to 13 years, found that exposure to cyberbullying predicts lower levels of empathic responsiveness over time (Pabian, Vandebosch, Poels, Van Cleemput, & Bastiaensens, 2016). These aforementioned studies suggest that extended exposure to cyberbullying can reduce empathy in adolescents, and individuals who are more impulsive are more likely to join in with the cyberbullying or ignore it. These findings support the assertion that cyberbystanders are complex with a range of characteristics. Given such complexity, there is a need to further understand cyberbystander subtypes in terms of their underlying neurobiology.

### 1.2 fMRI, aggression and cyberbullying

fMRI is a noninvasive imaging modality that provides an indirect measurement of brain activation by quantification of the hemodynamic response to a certain stimuli (Smith, 2004). Blood‐oxygenation‐level‐dependent (BOLD) task‐based fMRI (tb‐fMRI) has the capacity to measure hemodynamic responses to changing stimuli or task conditions with a reasonable spatial resolution by detecting the transient changes in deoxyhemoglobin concentration (Boynton, Engel, Glover, & Heeger, 1996; Buckner et al., 1996; Friston et al., 1998; Smith, 2004). Using tb‐fMRI, researchers can investigate brain regions and how these regions may be recruited to “process” different conditions and stimuli, such as witnessing the act of cyberbullying.

Research regarding the use of fMRI and cyberbullying is extremely limited and has mostly been addressed in conduct disorder studies. For example, fMRI has been employed in aggressive behavior in 16‐ to 18‐year‐old males (Decety, Michalska, Akitsuki, & Lahey, 2009), showing that youth with aggressive conduct disorder exhibit an atypical pattern of neural responses to viewing others in pain. For example, youth with conduct disorder showed activation in the insula and precentral gyrus, whereas control youth did not. The same researchers reported similar results in an earlier study regarding activity in regions of the brain in response to seeing others in pain (Decety, Michalska, & Akitsuki, 2008). From a structural perspective, a study of 9‐ to 18‐year‐old males and females found that callous‐unemotional traits are related to variations in brain structure (i.e., gray matter volume of the bilateral anterior insular cortices), but only in males (Raschle et al., 2018). Other research suggests that aggressive behavior might originate from an impairment of both recognition of stimuli that depict emotional valence and cognitive control of emotional behavior (Sterzer, Stadler, Krebs, Kleinschmidt, & Poustka, 2005), and that adolescents with aggressive behavior may also have significant differences in emotion processing and regulation networks (including orbitofrontal, dorsomedial prefrontal, and limbic cortex) (Raschle, Menks, Fehlbaum, Tshomba, & Stadler, 2015).

While these findings shed some light on how the developing brain responds to exclusion and aggression, information in relation to the brain mechanisms underlying cybervictims responses is lacking. No previous research has examined cyberbullying using fMRI in a real‐time situation such as observing a cyberbullying scenario or in a healthy, general population sample (i.e., nonconduct disorder, nonaggressive). Subsequently, the current study is the first to investigate cyberbullying using tb‐fMRI using a specific cyberbullying paradigm in healthy young people. The current pilot study has the following primary hypothesis: CyPicS tb‐fMRI will differentiate unique brain activations associated with cyberbullying compared to neutral conditions. More specifically, this research aims to address the following questions:

1. How does brain activation differ among individuals viewing cyberbullying versus neutral stimuli?
2. Does brain activation differ between groups with prior versus no experience of cyberbullying?

## 2 METHOD

This study was approved by the University of the Sunshine Coast, Human Research Ethics Committee.

### 2.1 Recruitment

Participants were recruited through the University of the Sunshine Coast. Information was shared about the study via social media posts, student newsletter, student support services, announcements in lectures, the university website, and word of mouth. Those who expressed interest in participating were sent the information sheet via email and were asked to contact the first author to book their study appointment. All participants gave their informed consent prior to their inclusion in the study. Exclusion criteria included those who suffer from a major neurological disorder, developmental disorder, intellectual disability, or major medical illness, had sustained a head injury (with loss of consciousness more than 30 min), or were deemed unsafe to undergo a magnetic resonance imaging (MRI) scan. Inclusion criteria included those who were aged between 18 and 25 years and were proficient in written and spoken English.

### 2.2 Participants

A total of 32 participants, aged 18 to 25 years, took part in the study. The mean age of the total sample was 21.56 ± 2.50 years and 65.6% (*n* = 21) were female.

### 2.3 Cyberbullying self‐report measure

As it was important to establish prior experiences of cyberbullying, the Berlin Cyberbullying‐Cybervictimization Questionnaire was used to assess previous experiences of cyberbullying and cybervictimization (Schultze‐Krumbholz & Scheithauer, 2009, 2011). Participants were asked if they had experienced a list of behaviors over a 6‐month period at any time in their life, as well as if they had acted in that way. The scale ranged from 0 (*has not happened to me at all*) to 4 (*several times a week*).

### 2.4 The Cyberbullying Picture Series

**Figure 1:**
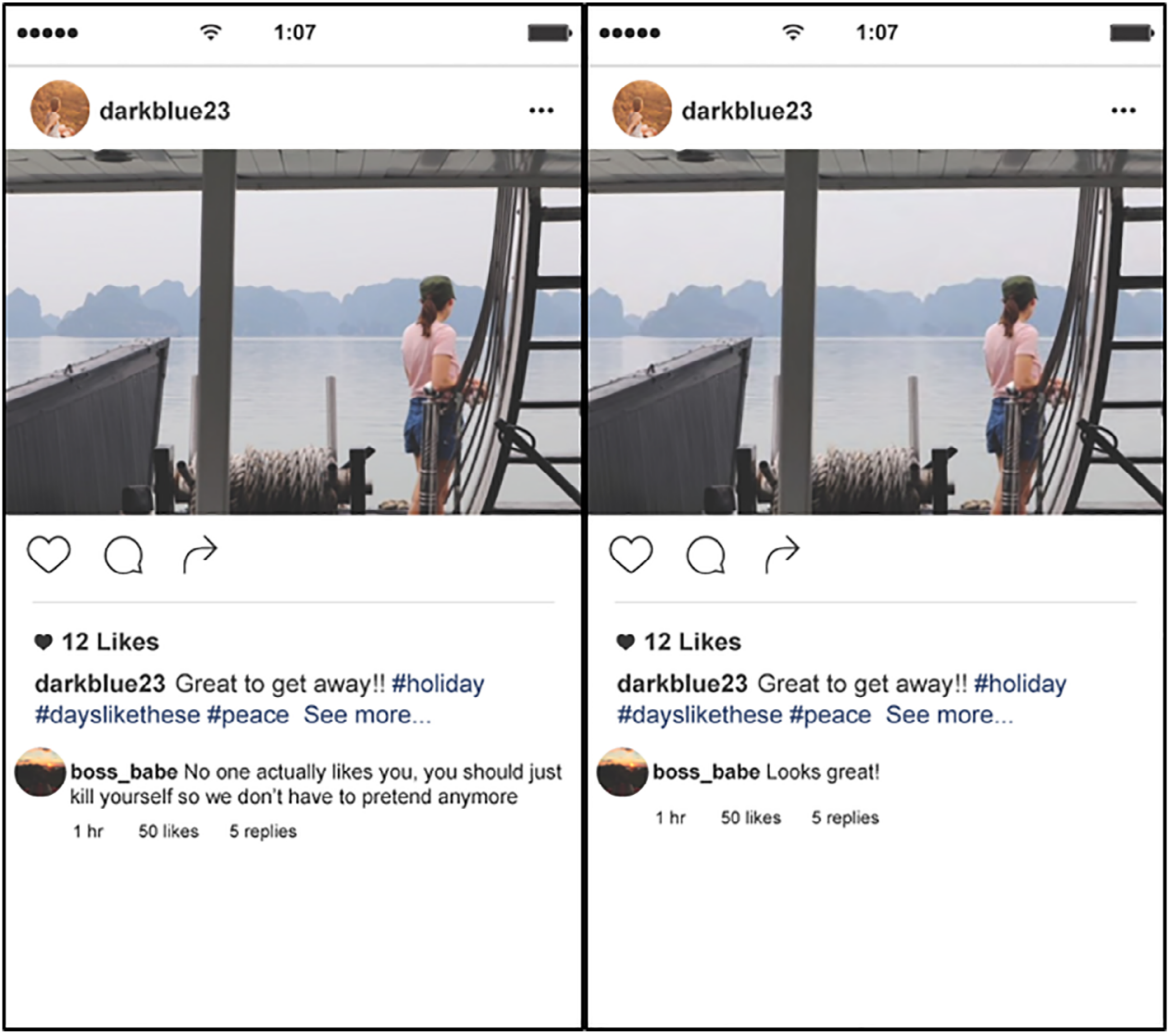
Example Cyberbullying (left) versus Neutral (right) stimuli from the Cyberbullying Picture Series (CyPicS)

A total of 12 scenarios were developed based on a single image used in the study by Bastiaensens et al. (2014). They were designed to appear as though they were images on a popular social networking site (with no branding of any particular site), with nuanced comments associated with them to determine their stimulus condition: cyberbullying or neutral. The cyberbullying comments were based on real life comments obtained from various social media platforms. The scenarios were designed to ensure an even distribution of female‐ and male‐based content and to ensure that the comments would be equally harsh for both genders. Each gender had a bullying comment associated with suicide attached to it, as this content is one of the more common and harmful forms of cyberbullying (Nilan, Burgess, Hobbs, Threadgold, & Alexander, 2015). Furthermore, each gender had a bullying comment associated with topical issues: body image for females (e.g., being overweight) and masculinity for males (e.g., targeting virginity). Importantly, the cyberbullying comment was given more “likes” than the post itself. This was to portray the sense of a power imbalance between the victim and bully, in that the victim would feel a sense of powerlessness when witnessing the cyberbullying comment receiving so many “likes.” Similarly, the sense of repetition, another criterion for cyberbullying, would have been felt, when seeing these “likes” continuing to rise on the cyberbullying comment. The images show no identifying information of the people depicted. Importantly, neutral stimuli contained the same image as the corresponding cyberbullying stimuli, including details such as “likes” pertaining to the image and the associated comment. Stimuli pairs (i.e., having the same images) were identical with the exception of the actual comment which differed in its valence. That is, a cyberbullying stimulus had a negative bullying comment, whereas a neutral stimulus had a neutral/positive comment (Figure 1).

These scenarios were presented as part of the experimental paradigm to form the Cyberbullying Picture Series (CyPicS) (a protocol paper describing the development of CyPicS has been submitted for publication, which details the development and validation of these scenarios). An important aspect of CyPicS development was that images used in the scenarios (without any comments) were assessed on the three dimensions of pleasure, arousal, and dominance using the Self‐Assessment Manikin (SAM) (Lang, 1980), and were compared to a similar selection of images from the International Affective Picture System (IAPS) with ranges of neutral to positive mean scores on the three dimensions (Lang, Bradley, & Cuthbert, 2008). IAPS was developed to “provide ratings of affect for a large set of emotionally‐evocative, internationally‐accessible, colour photographs that includes contents across a wide range of semantic categories” (Lang et al., 2008, p. 2). This process was conducted to ensure that responses to the cyberbullying stimuli were a result of the bullying comment rather than as an emotional response to the pictures; in other words, the valence of the stimulus is determined by the content of the comments and not the image itself.

Detailed results of the SAM ratings are included in the protocol paper, however, in summary, results indicated that the CyPicS images did not significantly differ on their ratings of valence. However, results indicated that the CyPicS sample found the CyPicS images to invoke less of an arousal emotion than the IAPS images. These results are supportive of using the CyPicS as a fMRI task, as researchers can be confident that the responses from participants will result from the associated cyberbullying comments rather than an emotional response to the image itself. Another aspect of the CyPicS development included rating each of the scenario's severity level and the six most severely rated scenarios were included in the fMRI task to ensure that an emotional response was evoked.

Furthermore, participants were asked upon conclusion of their scan about how realistic they found the images and how the images affected them. A list of resources (e.g., lifeline, beyondblue) was made available should they have required it.

### 2.5 fMRI design

**Figure 2:**
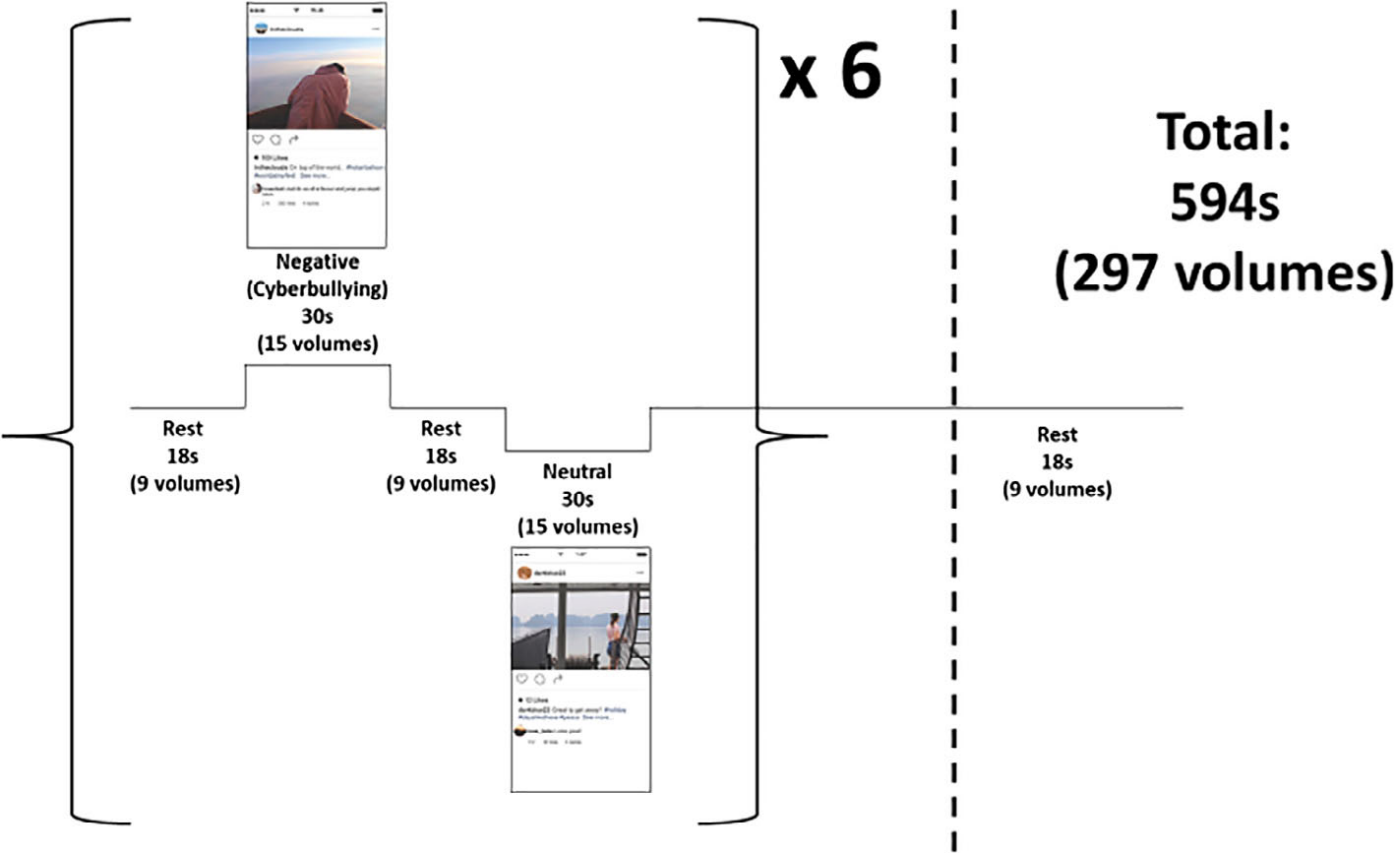
Task based functional magnetic resonance imaging (fMRI) design

Neural underpinnings of cyberbullying were assessed using the CyPicS task. Participants who evaluated CyPicS (McLoughlin et al., submitted) were ineligible to participate in the fMRI study. Thus, participants were asked to view six negative (cyberbullying) and six neutral stimuli while undertaking tb‐fMRI acquisition, with different captions to determine the stimulus condition. Each block consisted of approximately 30s activation (15 volumes) and 18s rest (nine volumes), and each stimulus was presented six times, totaling 594s (297 volumes). The stimuli included three images depicting a female and three images depicting a male (and each of these was duplicated across the two conditions). This design is summarized in Figure 2.

The CyPicS task was run via the commercially available software E‐Prime (v2.0) (Psychology Software Tools, 2018) and visualized within the scan room on a Magnetic Resonance Imaging (MRI) compatible NordicNeuro InroomViewingDevice (NordicNeuroLab, 2018), which was positioned outside the bore at the head end of the scanner. A reversed mirror was fitted to the 64‐channel head coil and adjusted after the participant was positioned at isocenter to ensure full view.

### 2.6 fMRI protocol

All scans were conducted on a 3‐Tesla Siemens Skyra MRI scanner (Erlangen, Germany) with a 64‐channel head and neck coil at the Sunshine Coast Mind and Neuroscience Thompson Institute (SCMNTI), University of the Sunshine Coast.

The MRI protocol consisted of a structural, whole‐brain three‐dimensional (3D) T1‐weighted Magnetisation‐Prepared Rapid‐Acquisition Gradient Echo sequence (MPRAGE; scan parameters time of repetition (TR) = 2,200 ms, echo time (TE) = 1.76 ms, inversion time (TI) = 850 ms, field of view (FOV) = 240 mm, 256 × 256 matrix, sagittal plane, spatial resolution = 0.9 mm isotropic, 208 slices, and scan duration = 4 min), which was optimized for gray/white matter contrast and used for the purpose of functional localization. Brain activation response to the CyPicS task was assessed using a T2*‐weighted multi slice EPI sequence (TR = 2000, TE = 30, FOV = 224 mm; 74 × 74 matrix, inplane resolution = 3 mm, IPAT6, simultaneous multislice (SMS) acceleration factor 3; transverse plane; slice thickness = 3 mm; 57 contiguous slices acquired top‐down, 297 volumes, scan duration = 9.54 min). Eighteen dummy scans were run (but no readout acquired) prior to the acquisition of the first TR readout. The CyPicS task was then automatically triggered from the first “true” RF pulse of the fMRI sequence. Prior to the tb‐fMRI sequence, a field map with the same FOV was acquired to aid in correcting image distortion due to field inhomogeneities. Self‐report measures assessed previous experiences of cyberbullying.

### 2.7 Data analysis

The fMRI analysis was performed using SPM12 (Wellcome Trust Centre for Human Neuroimaging, London, UK). Before processing, each participant's scans were checked for data quality; functional and structural data were visually inspected for artifacts, coverage of brain regions, and signal dropout. All MR image data passed the visual inspection of data quality and were included in the following analysis. The preprocessing of fMRI data included (a) 2‐pass motion correction in which fMRI volumes of each individual were aligned to the first volume, an average volume was created from aligned volumes, and then fMRI volumes were aligned to the average volume; (b) coregistration to the 3D anatomic image via the average fMRI image, and (c) normalization to the Montreal Neurological Institute (MNI) space via the anatomic image (Ashburner, Andersson, & Friston, 1999). Normalized volumes were smoothed with a 4 × 4 × 4 mm^3^ full width at half maximum Gaussian kernel using SPM12. No participants were excluded due to data quality or any other concerns.

The neural correlates of cyberbullying were analyzed using the two‐level general linear modeling (GLM) approach implemented in SPM12. At the subject level, the activation maps associated with each scenario were determined by correlating the BOLD response with the convolution of the hemodynamic response function (HRF) and stimulus (rest, cyberbullying, neutral) epochs defined by the stimulus‐on time and their durations (rest 18s, cyberbullying 30s, neutral 30s). A canonical HRF with time and dispersion derivatives was used. In the first level analysis, one statistical contrast, cyberbullying versus neutral scenes, was constructed for each participant. The contrast (difference in β) images of the first‐level analysis were then used for the second‐level group statistics to determine (a) neural correlates of cyberbullying across all participants (random effect analysis), (b) age‐related differences of brain response to cyberbullying (one‐sample regression analysis), (c) gender differences of brain response to cyberbullying (two‐samples *t* test), (d) differences of brain response to cyberbullying between groups of cyberbully/victim and victim groups, and (e) differences of brain response to cyberbullying between groups previously exposed to cyberbullying and group with no experience (two‐samples *t* test). Age was included as a covariate in the random effect analysis and all two‐samples tests. The significance of neural correlates were tested using family‐wise error (FWE) corrected cluster *p* value (*p*_*FWE*_ < .05) with a cluster forming voxel threshold of uncorrected *p* < .001.

## 3 RESULTS

### 3.1 Demographics

56.3% (*n* = 18) participants self‐identified as cyberbully‐victims, 34.4% (*n* = 11) as cybervictims, and 9.4% (*n* = 3) as noninvolved (i.e., had never experienced cyberbullying as a bully, victim, or both)

### 3.2 Cyberbullying versus neutral

**Figure 3:**
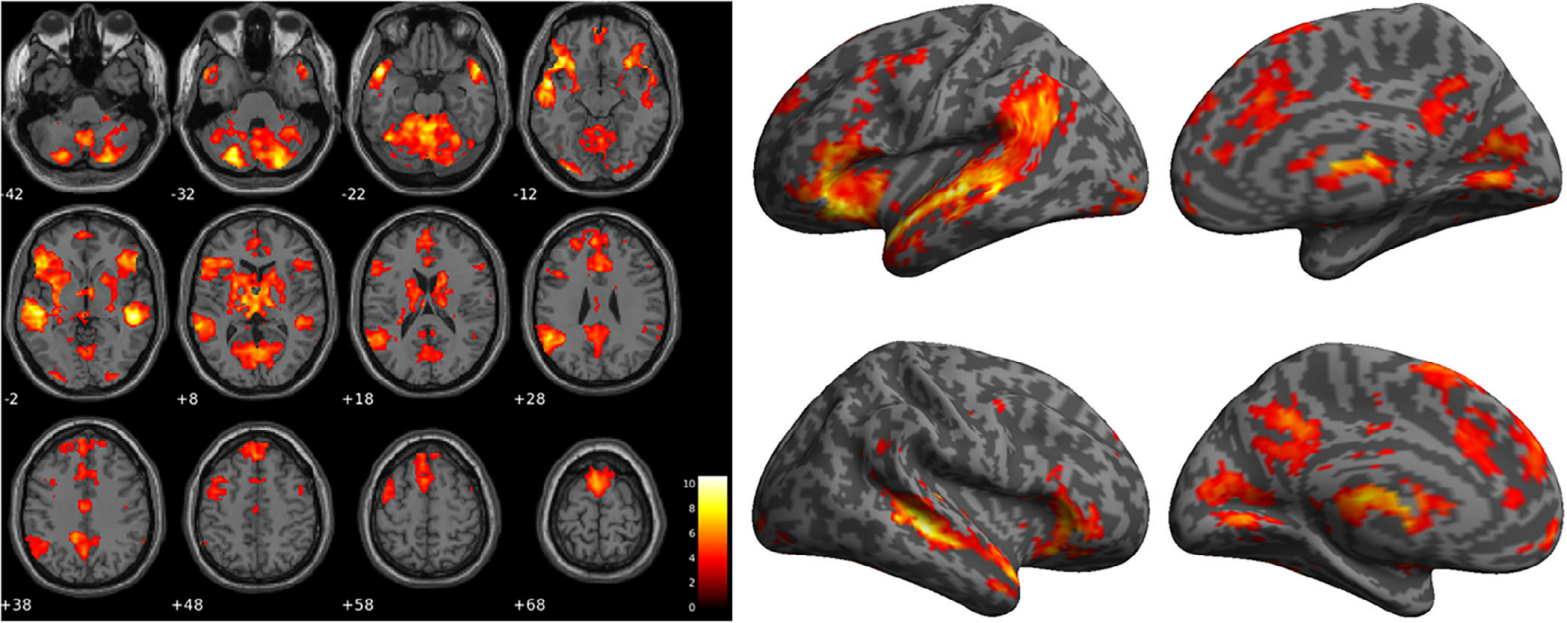
The mean activation map associated with cyberbullying images, that is, greater blood‐oxygenation‐level‐dependent (BOLD) viewing cyberbullying images than those viewing neutral scenes. Activation clusters were illustrated on selected axial slices on the left panel and the inflated brain surface on the right panel (top left: left lateral view; top right: medial view of the left hemisphere; bottom left right lateral view; bottom right: medial view of the right hemisphere). The color bar represents T statistic values. Large clusters diffuse across middle temporal gyrus, precuneus, angular gyrus, putamen, thalamus, inferior frontal gyrus, posterior cingulate cortex, cerebellum, lingual gyrus, anterior cingulate cortex, and superior frontal gyrus

The neural responses of witnessing cyberbullying (*p*_*FWE*_ < .05) are illustrated in Figure 3, including five large clusters. The first cluster (cluster level *p*_FWE_ < .001, cluster size *k*_E_ = 17,945, peak *T* value 10.62, peak Z scores 6.79, maximal MNI coordinate [−52 0–14]) diffuses across left (L‐) and right (R‐) middle temporal gyrus (MTG), precuneus, L‐ angular gyrus, L‐ and R‐ putamen, L‐ and R‐ thalamus, L‐ and R‐ inferior frontal gyrus (IFG). The second cluster (*p*_*FWE*_ < .001, *k*_*E*_ = 13,593, peak *T* value 9.6, peak Z scores 6.44, maximal MNI coordinate [−24–80 –32]) diffuses across L‐ and R‐ posterior cingulate cortex (PCC) and L‐ and R‐ posterior cerebellum. The third cluster (*p*_*FWE*_ < .001, *k*_*E*_ = 346, peak *T* value 7.1, peak Z scores 5.4, maximal MNI coordinate [−32–90 –12]) is in L‐ lingual gyrus. The fourth cluster (*p*_*FWE*_ < .001, *k*_*E*_ = 5,879, peak *T* value 6.92, peak Z scores 5.31, maximal MNI coordinate [−32–90 –12]) diffuses across L‐ and R‐ anterior cingulate cortex (ACC) and L‐ and R‐ superior frontal gyrus. The fifth cluster (*p*_*FWE*_ < .001, *k*_*E*_ = 287, peak *T* value 5.58, peak Z scores 4.58, maximal MNI coordinate [−2–14 38]) is in L‐ and R‐ caudodorsal ACC.

### 3.3 Age and gender differences

**Figure 4:**
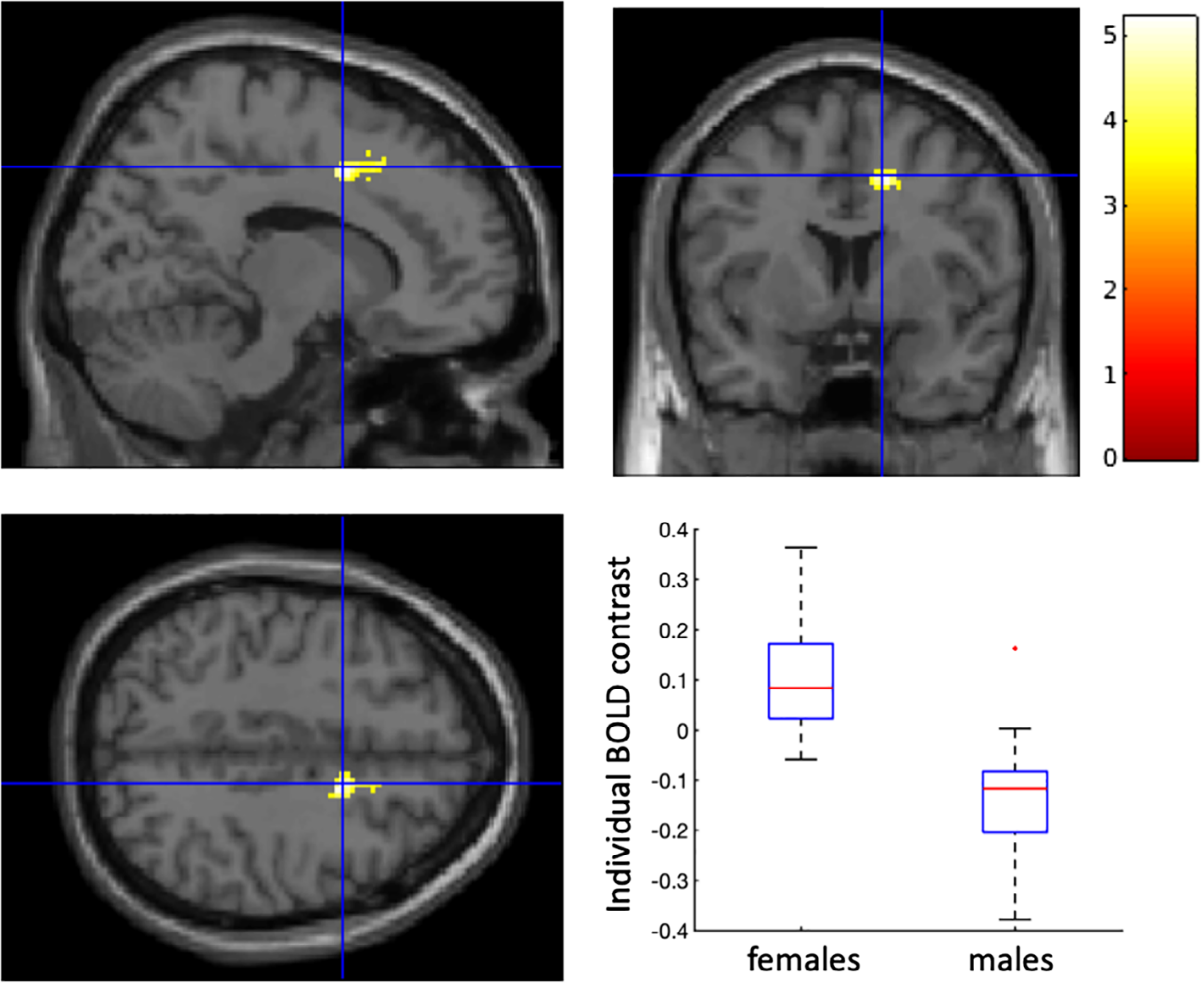
Greater activation in R‐ACC (cluster PFWE <.05 with a cluster forming voxel threshold of uncorrected *p* < .001, caudodorsal area 24, Montreal Neurological Institute (MNI) coordinates [14, 6, 44], cluster size 89 voxels) was observed in females compared to those in males when observing cyberbullying stimuli compared to neutral stimuli. The color bar represents T statistic values. The boxplot represents the averaged differences (contrast) in blood‐oxygenation‐level‐dependent (BOLD) signal changes in the cluster when a participant observing cyberbullying stimuli compared to neutral stimuli grouped by gender. On each box, the red central mark indicates the median, and the bottom and top edges of the box indicate the 25th and 75th percentiles, respectively. The outliers are plotted individually using the “+” symbol

There was no significant cluster (*p*_*FWE*_ > .05) showing age‐related differences of brain response to cyberbullying. However, female participants showed a greater BOLD response (*p*_*FWE*_ < .05) to cyberbullying in R‐ACC (caudodorsal area 24, MNI coordinates [14, 6, 44], cluster size 89 voxels) than male participants (Figure 4).

### 3.4 Prior experiences of cyberbullying

**Figure 5:**
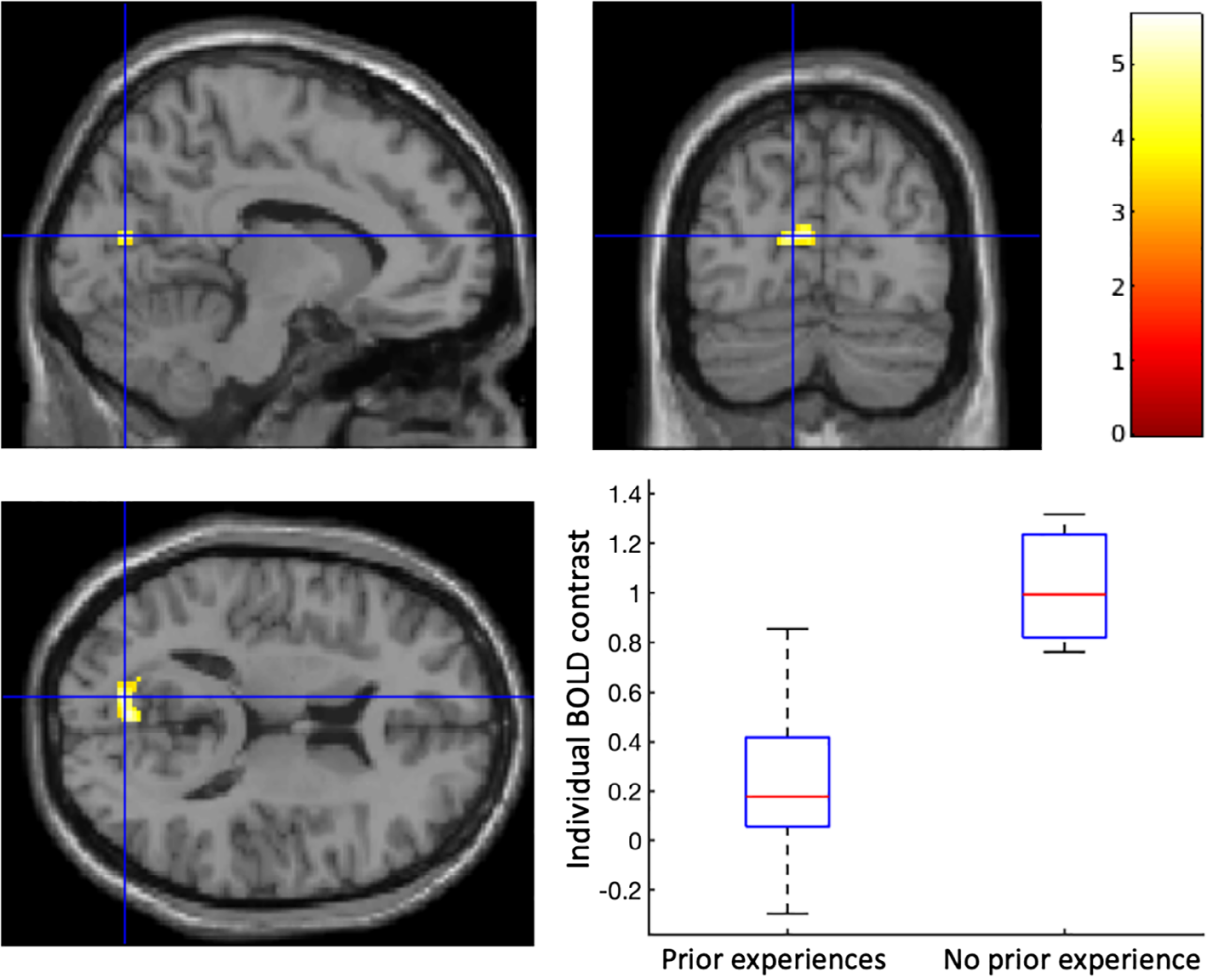
Greater activation in left precuneus (cluster PFWE <.05 with a cluster forming voxel threshold of uncorrected *p* < .001, MNI coordinates [−10, −76, 14], cluster size 117 voxels) was observed in those without prior experiences of cyberbullying compared to those with prior experiences when observing cyberbullying stimuli compared to neutral stimuli. The color bar represents T statistic values. The boxplot represents the averaged differences (contrast) in blood‐oxygenation‐level‐dependent (BOLD) signal changes in the cluster when a participant observing cyberbullying stimuli compared to neutral stimuli grouped by with/without prior experiences of cyberbullying. On each box, the red central mark indicates the median, and the bottom and top edges of the box indicate the 25th and 75th percentiles, respectively

There was no significant difference (*p*_*FWE*_ > .05) of brain response to cyberbullying between cyberbully‐victims and cybervictims, however, those with no prior experience of cyberbullying showed a greater BOLD response (*p*_*FWE*_ < .05) to cyberbullying in L‐Precuneus (MNI coordinates [−10, −76, 14], cluster size 117 voxels) (Figure 5), than those with any prior experience of cyberbullying (both cyberbully‐victims and cybervictims).

### 3.5 Participant feedback

Participants with no prior experience of cyberbullying reported finding the CyPicS harsher than those who had previously experienced cyberbullying. Similarly, female participants reported finding the CyPicS harsher than males. Interestingly, participants who had previously experienced cyberbullying noted that the CyPicS was not as harsh as what they themselves had experienced in the past. No participants reported feeling adversely affected by viewing the CyPicS, nor did any participants require a list of resources.

## 4 DISCUSSION

To our knowledge, this is the first article to report the neurobiological underpinnings of cyberbullying using fMRI to determine the neural responses of cyberbystanders. Our results revealed significant clusters of activation in response to cyberbullying stimuli compared to neutral stimuli across a distributed network of regions including L‐ and R‐ MTG. The MTG has been linked to cognition, including social cognition, suggesting that participants engaged areas regarding their social actions and emotions (Zahn et al., 2007). Clusters were also present in the L‐ and R‐ posterior cerebellum/vermis, traditionally linked with motion coordination, however, recent research suggests this is also linked with the processing of emotions such as happiness and disgust (Schienle & Scharmüller, 2013).

These findings are of particular interest, as social cognition is typically thought of as in conjunction with positive social interactions, and indeed there is evidence that those high in empathy are less likely to bully (Kokkinos & Kipritsi, 2012) whereas those lower in emotion recognition are more likely to bully (Lomas, Stough, Hansen, & Downey, 2012). Furthermore, victims of bullying have been found to demonstrate poor social cognition which may be linked to low levels of emotion perception and comprehension (Kokkinos & Kipritsi, 2012; Sutton, Smith, & Swettenham, 1999). Paradoxically, there is also evidence that high social intelligence is associated with antisocial behavior, such as bullying (Kaukiainen et al., 1999). Sutton et al. (1999) argue that bullying, particularly indirect bullying (exclusion, rumor‐mongering), requires insight into the victim's mental state to manipulate them (“theory of mind”). While the aforementioned literature shows substantive links between social cognition and cyberbullying, the current study is the first that draws links between social cognition and cyberbullying using fMRI. That is, the distinct pattern of BOLD response revealed by our study has provided further insights toward understanding the role of social cognition in the context of cyberbullying as evaluated by cyberbystanders.

Other areas activated included the L‐AG, one of the default mode network (DMN) hubs which is also associated with episodic memory (Thakral, Madore, & Schacter, 2017). This could indicate that participants were remembering their own past experiences of cyberbullying when observing these stimuli. The right superior temporal gyrus (R‐STG) also showed activation, and has been linked with experiencing emotional states such as sadness (Eugène et al., 2003), indicating an emotional response from participants when observing the cyberbullying stimuli. Other areas activated included the: right inferior frontal gyrus (R‐IFG), linked to self‐awareness (Goldberg, Harel, & Malach, 2006); precuneus, part of the DMN, associated with self‐evaluation and self‐consciousness (Kjaer, Nowak, & Lou, 2002); ACC, one of the DMN hubs, associated with empathy (Decety & Jackson, 2004; Jackson, Brunet, Meltzoff, & Decety, 2006); PCC, another DMN hub, activated by emotional stimuli, independent of valence (Maddock, Garrett, & Buonocore, 2003); thalamus, related to information relay; and putamen, identified as the hub of the “hate circuit” (Zeki & Romaya, 2008). These findings shed new light on the complexity of the brain responses in the witnessing of cyberbullying and suggest that there may be a complex series of emotion‐related processing occurring in cyberbystanders, including (potentially) sadness and self‐awareness, as well as hate and empathy.

Interestingly, our results showed that female participants had a greater BOLD response to the cyberbullying stimuli compared to neutral stimuli in the R‐ACC than males. As previously mentioned, the ACC plays a key role in the processing of empathy (Decety & Jackson, 2004; Jackson et al., 2006), but also emotional regulation (Stevens, Hurley, & Taber, 2011), possibly indicating that females may be more empathic cyberbystanders than males, or perhaps more able to regulate their emotions. Furthermore, results indicate those with no prior experience of cyberbullying showed a greater BOLD response to the cyberbullying stimuli compared to the neutral stimuli in the L‐Precuneus, the area of the brain responsible for self‐evaluation and self‐consciousness (Kjaer et al., 2002). One interpretation of this finding is that these participants may have imagined and evaluated their own, personal experience of the cyberbullying scenarios if they were the victim and/or bully. Furthermore, those with previous experiences of cyberbullying may have been habituated to the cyberbullying stimuli, and therefore have a blunted response in terms of self‐evaluation and/or self‐consciousness.

### 4.1 Limitations and future directions

There were several limitations associated with this study, which should be considered for future studies seeking to examine the neural responses linked to cyberbullying. First, as past research has shown that a victim's response to the cyberbully (whether angry, sad, or confident) may influence the bystander's perceived seriousness of the cyberbullying (Sokol, Bussey, & Rapee, 2015) future studies should build on our protocol and investigate how the subsequent response by the “cybervictim” influences the cyberbystanders reactions to these scenarios. Second, given the small sample size of this pilot study, our comparison of each gender and of those with versus those without previous experience of cyberbullying should be interpreted with some caution. Future research should seek to replicate this with larger samples. Third, this study is limited to the social media platform depicted in CyPicS. Future research should examine other forms of cyberbullying via alternate platforms, as well as other forms of cyberbullying such as exclusion. In addition, future research should examine participants' views on the likelihood of these scenarios occurring in real life (and whether this could influence BOLD response), as well as any changes in mood pre and post scan.

Furthermore, given that most research to date has examined the developing brain, and because of the pilot nature of this study, young adults were chosen for feasibility reasons. However, we plan to replicate this protocol to be implemented in adolescents, especially given that this pilot data has demonstrated a significant effect. Given the considerable amount of overlap and circularity between traditional bullying and cyberbullying (Zych, Ortega‐Ruiz, & Del Rey, 2015), and that the combination of both traditional bullying and cyberbullying has the most negative/severe impact on mental health (Landstedt & Persson, 2014), further research examining both forms of bullying and how the brain responds would be valuable.

Future studies should formally assess social cognition to disentangle the neural responses of social cognition from those generated by cyberbullying, with particular attention on whether empathy mediates any responses to witnessing cyberbullying. In addition, future research could examine whether scores on additional measures such as social cognition, well‐being, personality traits, or emotional state is predictive of activation in the cyberbullying condition. Similarly, resting state data could identify whether those that have been exposed to cyberbullying in the past have altered resting state connectivity in social cognition networks, compared to those who have no prior experience of cyberbullying. Finally, while the focus of this pilot study was to demonstrate that the cyberbullying stimuli elicited a different BOLD response to that of the neutral stimuli, future studies could also consider evaluating whether the valence or severity of individual cyberbullying scenarios elicited increased BOLD responses.

## 5 CONCLUSION

Our article has highlighted that there are a range of brain regions activated in cyberbystanders when observing cyberbullying stimuli, reflecting a “socio‐emotional/self‐referential” network. Furthermore, we found significant differences in the pattern of responses between males compared to females, as well as in those with versus those without prior experience of cyberbullying. Overall, our findings make an important contribution to the extant literature and provide new insights as to the way cyberbystanders respond (neurobiologically) to different cyberbullying stimuli conditions. Finally, this study demonstrates that the neurobiological underpinnings of cyberbullying can be examined using fMRI, and future research should focus on how this can be further developed and then understood to inform education programs as well as interventions that may improve outcomes for those who are at risk or vulnerable to mental health conditions.

## CONFLICT OF INTERESTS

The authors have no conflict of interest to declare.

## Data Availability

The data that support the findings of this study are available on request from the corresponding author. The data are not publicly available due to privacy or ethical restrictions.
